# Genetic Screen in Chlamydia muridarum Reveals Role for an Interferon-Induced Host Cell Death Program in Antimicrobial Inclusion Rupture

**DOI:** 10.1128/mBio.00385-19

**Published:** 2019-04-09

**Authors:** Amanda M. Giebel, Shuai Hu, Krithika Rajaram, Ryan Finethy, Evelyn Toh, Julie A. Brothwell, Sandra G. Morrison, Robert J. Suchland, Barry D. Stein, Jörn Coers, Richard P. Morrison, David E. Nelson

**Affiliations:** a Department of Microbiology and Immunology, Indiana University School of Medicine, Indianapolis, Indiana, USA; b Department of Molecular Genetics and Microbiology, Duke University Medical Center, Durham, North Carolina, USA; c Department of Microbiology and Immunology, University of Arkansas for Medical Sciences, Little Rock, Arkansas, USA; d Division of Allergy and Infectious Disease, Department of Medicine, University of Washington, Seattle, Washington, USA; e Department of Biology, Indiana University, Bloomington, Indiana, USA; f Department of Immunology, Duke University Medical Center, Durham, North Carolina, USA; University at Buffalo; UTHSCSA; Rocky Mountain Laboratories, National Institute of Allergy and Infectious Diseases

**Keywords:** *Chlamydia*, host-pathogen interactions, interferon-stimulated genes, intracellular pathogens, molecular genetics

## Abstract

Interferon-regulated immune defenses protect mammals from pathogenically diverse obligate intracellular bacterial pathogens of the genus Chlamydia. Interferon gamma (IFN-γ) is especially important in controlling the virulence of Chlamydia species and thus impacts the modeling of human chlamydial infection and disease in mice. How IFN-γ contributes to cell-autonomous defenses against Chlamydia species and how these pathogens evade IFN-γ-mediated immunity in their natural hosts are not well understood. We conducted a genetic screen which identified 31 IFN-γ-sensitive (Igs) mutants of the mouse model pathogen Chlamydia muridarum. Genetic suppressor analysis and lateral gene transfer were used to map the phenotype of one of these mutants, Igs4, to a missense mutation in a putative chlamydial inclusion membrane protein, TC0574. We observed the lytic destruction of Igs4-occupied inclusions and accompanying host cell death in response to IFN-γ priming or various proapoptotic stimuli. However, Igs4 was insensitive to IFN-γ-regulated cell-autonomous defenses previously implicated in anti-Chlamydia trachomatis host defense in mice. Igs4 inclusion integrity was restored by caspase inhibitors, indicating that the IFN-γ-mediated destruction of Igs4 inclusions is dependent upon the function of caspases or related prodeath cysteine proteases. We further demonstrated that the Igs4 mutant is immune restricted in an IFN-γ-dependent manner in a mouse infection model, thereby implicating IFN-γ-mediated inclusion destruction and host cell death as potent *in vivo* host defense mechanisms to which wild-type C. muridarum is resistant. Overall, our results suggest that C. muridarum evolved resistance mechanisms to counter IFN-γ-elicited programmed cell death and the associated destruction of intravacuolar pathogens.

## Abstract (precis)

Multiple obligatory intracellular bacteria in the genus Chlamydia are important pathogens. In humans, strains of C. trachomatis cause trachoma, chlamydia, and lymphogranuloma venereum. These diseases are all associated with extended courses of infection and reinfection that likely reflect the ability of chlamydiae to evade various aspects of host immune responses. Interferon-stimulated genes, driven in part by the cytokine interferon gamma, restrict the host range of various Chlamydia species, but how these pathogens evade interferon-stimulated genes in their definitive host is poorly understood. Various Chlamydia species can inhibit death of their host cells and may have evolved this strategy to evade prodeath signals elicited by host immune responses. We present evidence that chlamydia-induced programmed cell death resistance evolved to counter interferon- and immune-mediated killing of Chlamydia-infected cells.

## INTRODUCTION

Obligate intracellular bacteria in the genus Chlamydia, including the human pathogen Chlamydia trachomatis, undergo a developmental cycle in which they transition between infectious elementary body (EB) and noninfectious replicative reticulate body (RB) forms. Immune-mediated changes in host cell metabolism, including the degradation of intracellular tryptophan by the interferon gamma (IFN-γ)-inducible enzyme indole-2,3-dioxygenase, prompt physiological changes in C. trachomatis. For example, tryptophan starvation causes RBs to transition into nonreplicative aberrant forms, which persist without dividing or transitioning into EBs (1, 2). Under tryptophan starvation conditions, C. trachomatis genital strains express a partial tryptophan operon enabling the synthesis of tryptophan from indole, which may be derived from the genital microbiome, and are thereby able to survive in a tryptophan-depleted intracellular environment (3–6). C. trachomatis and Chlamydia muridarum can also block cell death in epithelial cells exposed to prodeath signals (7, 8). These observations suggest that Chlamydia spp. counter intracellular immune defenses while maintaining the viability of their host cell to complete a productive developmental cycle. However, the pertinent host defenses, including the trigger of prodeath pathways and the corresponding immune evasion mechanisms employed by different Chlamydia spp. remain largely unexplored.

The Th1 cytokine interferon gamma (IFN-γ) plays a central role in the immune protection of mice and humans against Chlamydia spp. (9). IFN-γ induces the expression of interferon-stimulated genes (ISGs), which mediate cell-autonomous defenses that clear intracellular pathogens (9–11). Multiple ISG-mediated cell-autonomous host defenses protect mice against experimental C. trachomatis infection (3, 9, 12–18). In contrast to C. trachomatis, mice are significantly more susceptible to infection and disease mediated by the rodent-adapted pathogen C. muridarum, presumably because C. muridarum encodes host-tailored virulence factors that help it counter the relevant IFN-γ-regulated cell-autonomous defenses of its definitive murine host (5, 16). Identification of the virulence factors that C. muridarum and C. trachomatis use to circumvent conserved and host-specific IFN-γ-regulated cell-autonomous host defense mechanisms could provide insight into mechanisms of chlamydial pathogenesis and guide the development of improved mouse models of C. trachomatis infection and disease (5, 19).

Increasing evidence suggests that programmed cell death resistance (PCDR) elicited by Chlamydia spp. is an ancient virulence strategy that contributes to chlamydial pathogenesis. Chlamydia-infected cells resist killing by a various synthetic and immune stimuli that elicit apoptosis of uninfected cells *in vitro* (7, 8, 20, 21), and PCDR may protect Chlamydia*-*infected cells from immune-mediated death signals (22, 23). The microbe’s ability to maintain integrity of its inclusion membrane is critical for the survival of C. trachomatis-infected host cells. Loss-of-function mutations in several inclusion membrane proteins (Incs; e.g., CpoS) prompt inclusion lysis and trigger cell death emanating from the destabilized inclusion (24, 25). Infection with Inc mutants deficient for inclusion maintenance triggers host cell death dependent on the autophagy protein Beclin1 and the cytosolic immune sensor stimulator of interferon genes (STING) but independent of the STING-mediated type I IFN response (24). Thus, the loss of inclusion integrity induces death pathways that override chlamydial PCDR. While Inc deficiencies can destabilize inclusions, host cell priming with IFN-γ can induce the disruption of inclusions formed by wild-type C. trachomatis inside mouse fibroblasts (26). Thus, an IFN-γ-mediated host response can override chlamydial PCDR, albeit only in mouse cells infected with C. trachomatis. However, neither the IFN-γ-activated host response leading to inclusion disruption nor the chlamydial factors counteracting this membranolytic host defense program have been identified.

The goal of this study was to identify C. muridarum genes that counter IFN-γ-regulated cell-autonomous defenses in mice. We identified interferon gamma-sensitive (Igs) C. muridarum mutants using a genetic screen. IFN-γ-mediated killing of one of these mutants, Igs4, was linked to an amino acid substitution in a putative chlamydial Inc protein. Interferon priming and proapoptotic stimuli compromised Igs4 inclusions and killed Igs4-infected mouse cells. Caspase inhibitors blocked both host cell death and lysis of Igs4 inclusions in IFN-γ-primed cells, suggesting that prodeath cysteine proteases operate upstream of inclusion lysis. Importantly, attenuation of Igs4 in wild-type mice was reversed in IFN-γ knockout (*IFN-γ* ^−/−^) mice, demonstrating the relevance of our *in vitro* observations in a model that mimics human genital chlamydia. Overall, our results show that chlamydial PCDR and interferon resistance are linked and suggest that C. muridarum has evolved mechanisms to protect inclusions against an IFN-γ-triggered membranolytic pathway executed by prodeath cysteine proteases.

## RESULTS

### Isolation of IFN-γ-sensitive Chlamydia muridarum mutants.

C. trachomatis and C. muridarum infect a variety of mouse epithelial and fibroblastic cell lines, including McCoy fibroblasts (McCoy cells) (3, 16). However, low doses of IFN-γ block C. trachomatis but not C. muridarum proliferation in McCoy cells, indicating that C. muridarum is equipped with virulence factors that counteract host defenses active in IFN-γ-primed mouse cells. To identify these factors, we designed a genetic screen comparing the inclusion formation of wild-type C. muridarum and 2,976 ethyl methanesulfonate (EMS)-mutagenized C. muridarum isolates in IFN-γ-primed and unprimed McCoy cells.

**FIG 1:**
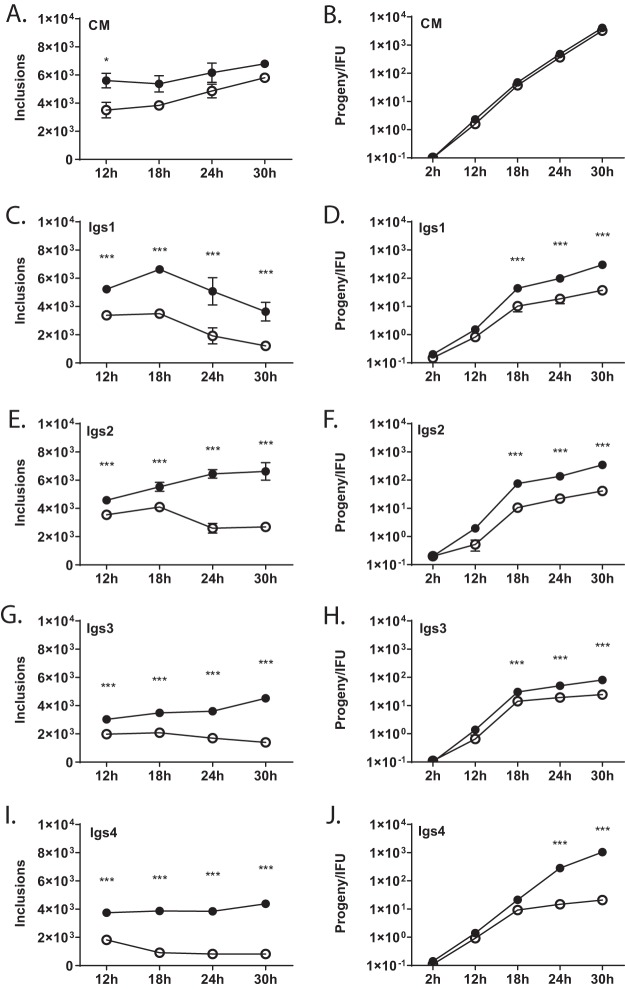
Development of Igs mutants is inhibited by IFN-γ. McCoy cells were infected at an MOI of 1 with *C. muridarum* (CM) or Igs mutants (indicated in graphs) ± IFN-γ. Inclusion-forming units (A, C, E, G, and I) and progeny (B, D, F, H, and J) were counted at different intervals postinfection. The *y* axis in the progeny experiments indicates burst size (number of inclusions counted in the progeny relative to the input inclusion-forming units in the original infection). Graphs show results of three experiments performed in triplicate, and error bars show standard deviation. −IFN-γ, closed circles; +IFN-γ, open circles. Corresponding infections ± IFN-γ were compared by two-way ANOVA with Bonferroni posttest. *, P < 0.05; ***, P < 0.001.

IFN-γ reduced C. muridarum inclusion formation, a measure of infectivity, in McCoy cells by approximately 20% (Fig. 1A). We identified 31 IFN-γ-sensitive (Igs) C. muridarum mutants that were more sensitive to IFN-γ than wild-type C. muridarum. Temporal inclusion and progeny assays were used to characterize four mutants, Igs1 to Igs4 (Fig. 1). IFN-γ strongly inhibited progeny production of these Igs mutants at 18, 24, and 30 h postinfection (hpi) (*P* < 0.001) (Fig. 1C to J). IFN-γ almost completely abrogated progeny production of Igs4 (Fig. 1J), and the number of Igs4 genomes began to decline between 18 and 24 hpi (see Fig. S1 in the supplemental material), suggesting that IFN-γ induced killing of this mutant in McCoy cells.

10.1128/mBio.00385-19.1 FIG S1 IFN-γ elicits killing of Igs4 in McCoy cells. McCoy cells ± IFN-γ were infected at an MOI of 1 with *C. muridarum* (CM) or Igs4, and DNA was harvested from the infected cells at various intervals postinfection. Quantitative real-time PCR was performed using a primer-probe set against the *C. muridarum* 16S rRNA gene, and genome copy numbers were calculated by comparing the amplification curves to dilutions of a plasmid with a cloned copy of *C. muridarum* 16S rRNA. The results are from a representative experiment performed in triplicate. Download FIG S1, PDF file, 0.5 MB. Copyright © 2019 Giebel et al. 2019 Giebel et al. This content is distributed under the terms of the Creative Commons Attribution 4.0 International license.

### An amino acid substitution in an inclusion membrane protein confers Igs4 IFN-γ sensitivity.

**FIG 2:**
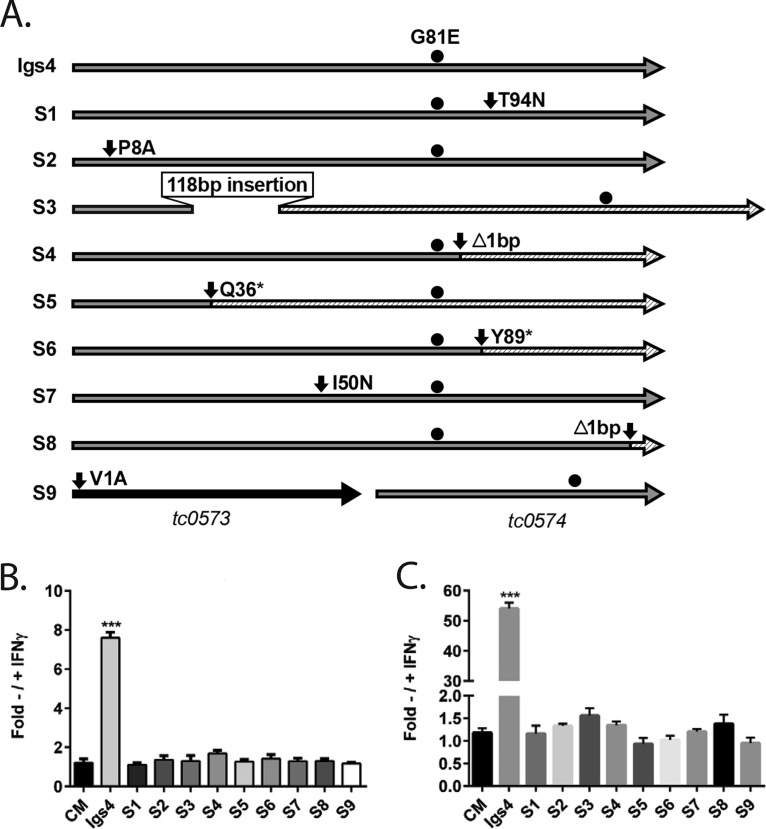
Igs4 IFN-γ sensitivity is linked to a G81E substitution in TC0574. (A) Schematic of Igs4 TC0574^G81E^ and predicted amino acid changes in TC0573 and TC0574 in suppressor mutants S1 to S9. S9 is scaled differently to include TC0573. All of the suppressor mutants retained TC0574^G81E^ (black circle) from Igs4. Hatched areas indicate untranslated regions of TC0574 caused by nonsense mutations (*), out-of-frame insertions (ins), or frameshifts (Δ). Ratio of inclusion (B) and progeny production (C) of *C. muridarum* (CM), Igs4, and S1 to S9 ± IFN-γ. Graphs show results from three experiments performed in triplicate, and error bars show standard deviation. The ratios of Igs4 to S1 to S9 inclusion and progeny production ± IFN-γ were compared to *C. muridarum* by two-way ANOVA with Bonferroni posttest. ***, P < 0.001.

We compared the genomes of several Igs mutants, but the relatively high mutation loads in the mutants and the various strengths of their IFN-γ-sensitivity phenotypes confounded attempts to identify the relevant mutations (Table S1). As an alternative strategy to identify causative mutations, we serially passaged Igs4 in IFN-γ-primed McCoy cells to isolate revertants whose resistance to IFN-γ-induced cell-intrinsic immunity was restored in mouse cells. Genome resequencing of select revertants revealed that most of them were isogenic to Igs4, except for new mutations in *tc0574* (Table S1). Since Igs4 carries a mutant *tc0574* allele (*tc0574^G242A^*) that introduces a nonsynonymous amino acid change in TC0574 (TC0574^G81E^), this indicated that the new mutations in *tc0574* of the revertants suppressed the effects of the mutant *tc0574* allele in Igs4 (Fig. 2). Targeted sequencing of the *tc0574* locus identified new mutations in *tc0574* in 22 of the isolates (8 unique genotypes, S1 to S8) and a point mutation that destroyed the predicted start codon of the upstream gene *tc0573* in one isolate (S9) (Fig. 2A). None of these mutations were silent, and 2 of the 9 mutations introduced stop codons in *tc0574*, showing that destroying TC0574^G81E^ mutant protein or preventing its expression suppressed the IFN-γ sensitivity conferred by the original mutation. Since inclusion and progeny production of the Igs4 suppressor mutants were similar to C. muridarum in IFN-γ-primed cells, this also showed that wild-type TC0574 is normally dispensable for C. muridarum IFN-γ resistance (Fig. 2B and C). We confirmed that *tc0574^G242A^* caused Igs4 IFN-γ-sensitivity using counterselection lateral gene transfer (27, 28). IFN-γ-resistant recombinants from a cross of Igs4 with a temperature-sensitive C. muridarum mutant retained wild-type *tc0574*, and one recombinant, R5, retained all the nonsynonymous alleles in Igs4 except for *tc0574^G242A^* (Fig. S2). We concluded that a detrimental missense mutation in a putative inclusion membrane protein (Inc) gene *tc0574*, which is normally dispensable for C. muridarum IFN-γ resistance, sensitizes Igs4 to IFN-γ.

10.1128/mBio.00385-19.2 FIG S2 Igs4 IFN-γ sensitivity is linked to a G81E substitution in TC0574. (A) Nonsynonymous amino acid changes in Igs4, R3, R5, and R7 compared to *C. muridarum* (CM). Percent intact inclusions for *C. muridarum*, temperature-sensitive *C. muridarum* (CM^TS^), Igs4, *C. muridarum* (CT), and recombinants R3, R5, and R7 at 37°C (B) and 40°C (C) ± IFN-γ. Graphs show results from three experiments performed in triplicate, and error bars show standard deviation. Corresponding ± IFN-γ infections were compared by two-way ANOVA with Bonferroni posttest. ****, *P* < 0.0001. Strains that did not grow under the indicated condition are denoted with a pound sign (#). Download FIG S2, PDF file, 0.1 MB. Copyright © 2019 Giebel et al. 2019 Giebel et al. This content is distributed under the terms of the Creative Commons Attribution 4.0 International license.

10.1128/mBio.00385-19.8 TABLE S1 Single-nucleotide polymoprhisms in the genomes of IGS mutants. Download Table S1, DOCX file, 0.3 MB. Copyright © 2019 Giebel et al. 2019 Giebel et al. This content is distributed under the terms of the Creative Commons Attribution 4.0 International license.

### Mechanisms of Igs4 and C. trachomatis IFN-γ sensitivity differ.

**FIG 3:**
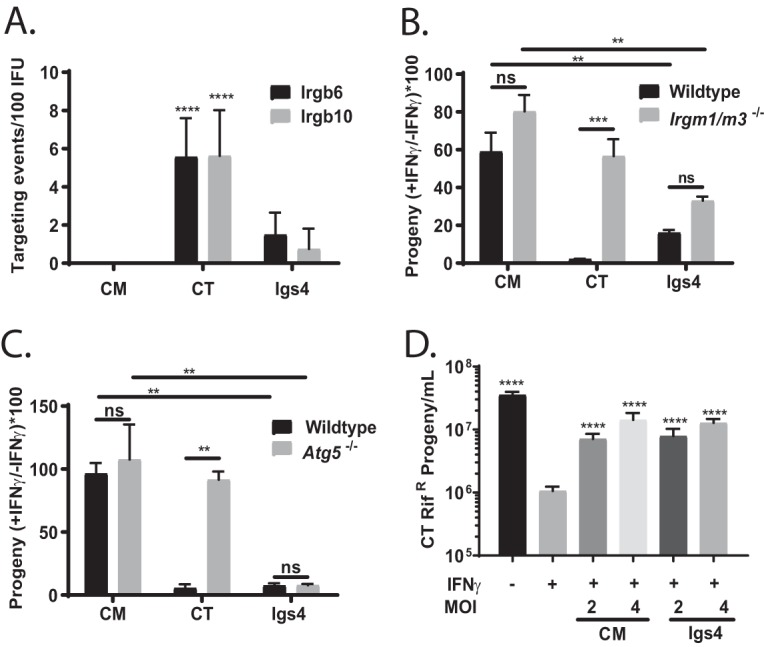
Igs4 resists IRGs that inhibit *C. trachomatis* in mouse cells. (A) Irgb6 and Irgb10 targeting events per 100 inclusions in *C. muridarum* (CM), *C. trachomatis* (CT), and Igs4-infected McCoy cells plus IFN-γ; ****, *P* < 0.0001. (B and C) *irgm1*^−/−^ or *irgm3*^−/−^ (B) or *atg5^−/−^* (C) MEFs were infected with *C. muridarum*, *C. trachomatis*, or Igs4 ± IFN-γ, and the ratios of progeny the strains that produced ± IFN-γ were compared. **, P < 0.01; ***, *P* < 0.001; ns, not significant. (D) McCoy cells were infected with *C. trachomatis* rif^r^ at an MOI of 1 or coinfected with *C. trachomatis* rif^r^ at an MO1 and Igs4 or *C. muridarum* at MOIs of 2 or 4 ± IFN-γ (indicated below bars). Rifampin was added in all experiments to inhibit *C. muridarum* and Igs4. *C. trachomatis* rif^r^ progeny were harvested at 24 hpi and counted in McCoy cells. Graph shows the results from three experiments performed in triplicate, and error bars indicate standard deviation. Progeny under various conditions were compared to *C. trachomatis* rif^r^ plus IFN-γ by one-way ANOVA with Bonferroni posttest. ****, P < 0.001.

In IFN-γ-primed mouse fibroblasts, C. trachomatis inclusions are targeted by GKS class immunity-related guanosine phosphatases (IRGs) that promote inclusion disruption and elimination of the bacteria, likely via autolysosomes (12, 15, 29). To test whether Igs4 is sensitive to IFN-γ-mediated cell-autonomous defenses that restrict C. trachomatis in mouse cells, we compared the subcellular localization of two GSK IRGs, Irgb10 and Irgb6, in cells infected with C. trachomatis, C. muridarum, or Igs4. Irgb6 and Irgb10 localized to a subpopulation of C. trachomatis, but not C. muridarum or Igs4, inclusions, suggesting that Igs4 is not susceptible to an IRG-dependent host defense pathway (Fig. 3A). We also tested if Igs4 progeny production was restored in cells that lack Irgm1 and Irgm3, two regulatory proteins that mediate Irgb6 and Irgb10 delivery to inclusions (30). Progeny production of C. trachomatis, but not of Igs4, increased in IFN-γ-treated *irgm1*^−/−^ or *irgm3*^−/−^ murine embryonic fibroblasts (MEFs), confirming that IFN-γ-mediated inhibition of Igs4 is independent of IRGs (Fig. 3B). The autophagy protein Atg5 is essential for the delivery of IRGs and related interferon-inducible guanylate binding proteins (GBPs) to C. trachomatis inclusions and the execution of antimicrobial xenophagy (26, 30–32). Igs4 formed similar numbers of inclusions in IFN-γ-primed *atg5*^−/−^ and in wild-type MEFs, excluding the involvement of autophagy or autophagy-related processes in the defense pathway that is effective against Igs4 (Fig. 3C). Finally, C. muridarum EBs can rescue C. trachomatis progeny production and block the recruitment of IRGs to C. trachomatis inclusions in IFN-γ-primed mouse cells (15, 33). Igs4 and C. muridarum similarly rescued C. trachomatis in coinfection rescue experiments in IFN-γ-primed McCoy cells (Fig. 3D). These data demonstrated that Igs4 is sensitive to an IRG- and Atg5-independent IFN-γ-inducible defense program and that Igs4 differs in this regard from IRG-sensitive C. trachomatis.

**FIG 4:**
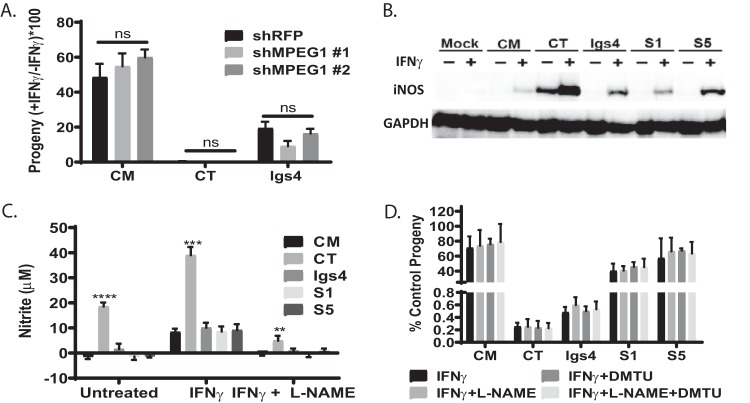
Igs4 resists non-IRG IFN-γ defenses that inhibit other *Chlamydia* spp. (A) Mpeg1 transcript was knocked down >70% in MEFs utilizing shRNA MPEG#1 or MPEG #2. The ratios of the progeny *C. muridarum* (CM), *C. trachomatis* (CT), or Igs4 produced in ± IFN-γ at 30 hpi in knockdown MEFs were compared. ns, not significant. (B) iNOS and GAPDH Western blot of mock-, *C. muridarum*-, *C. trachomatis*-, Igs4-, S1-, or S5-infected McCoy cells ± IFN-γ (indicated above lanes) at 24 hpi. (C) Nitrite levels in supernatants of McCoy cells infected with *C. muridarum*, *C. trachomatis*, Igs4, S1, or S5 ± IFN-γ and l-NAME at 24 hpi; **, P < 0.01; ***, P < 0.001; ****, P < 0.0001. (D) Progeny production of *C. muridarum*, *C. trachomatis*, Igs4, S1, or S5 in untreated McCoy cells (control) compared to McCoy cells treated with IFN-γ, IFN-γ plus l-NAME, IFN-γ plus dimethyl thioureau (DMTU), or IFN-γ plus l-NAME plus DMTU at 24 hpi. Error bars indicate standard deviations, and data were analyzed by two-way ANOVA with Bonferroni posttest. None of the differences were significant.

We tested if Igs4 was sensitive to other non-IRG IFN-γ-mediated defenses that can contribute to chlamydia clearance. For example, macrophage-expressed protein 1 (Mpeg1) contributes to killing of various Chlamydia spp. in fibroblasts (34). However, we observed that C. trachomatis and Igs4 produced similar numbers of progeny in IFN-γ-primed wild-type MEFs and in MEFs in which Mpeg1 was knocked down using short hairpin RNAs (shRNAs) (Fig. 4A). IFN-γ also induces the production of nitric oxide and reactive oxygen species that can kill C. trachomatis (17, 18, 35, 36). C. trachomatis elicited higher levels of inducible nitric oxide synthase (iNOS) expression and nitrite production than did C. muridarum or Igs4 in McCoy cells (Fig. 4B and C). However, Igs4 progeny production was not rescued by the iNOS inhibitor N(G)-nitro-l-arginine methyl ester (l-NAME) or the oxygen scavenger dimethylthiourea (DMTU) in IFN-γ-treated McCoy cells (Fig. 4D). These results showed that different IFN-γ-mediated defenses contribute to the inhibition of C. trachomatis and Igs4 in mouse cells.

### Type I and type II interferons elicit lysis of Igs4 inclusions in mouse cells.

**FIG 5:**
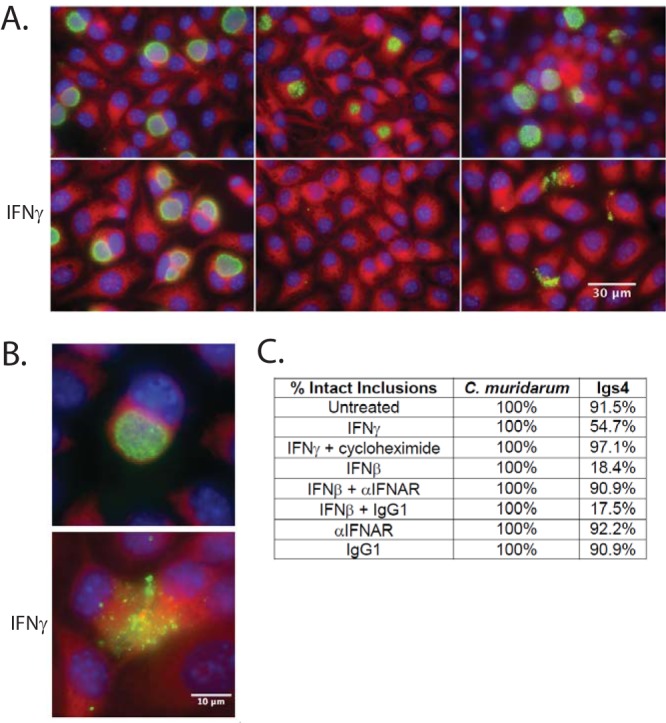
Type I and II interferons elicit lysis of Igs4 inclusions. (A) McCoy cell monolayers were infected with *C. muridarum* (CM), *C. trachomatis* (CT), or Igs4 at an MOI of 0.1 ± IFN-γ. The infected cells were fixed at 24 hpi and labeled with anti-LPS (green), DAPI (blue), and Evans blue (red). (B) Representative Igs4 inclusions labeled as described in panel A. (C) Mean percent intact inclusions in IFN-γ-, IFN-β-, α-IFNAR-, α-IFNAR plus IFN-β-, or CHX-treated McCoy cells at 24 hpi from two experiments performed in triplicate.

Lipopolysaccharide (LPS) staining revealed lysed Igs4 inclusions in untreated McCoy cells and that the number of these lysed inclusions dramatically increased by 16 hpi in IFN-γ-primed cells. In contrast, lysed inclusions were rare in McCoy cells infected with C. muridarum, C. trachomatis, other Igs mutants, or isogenic Igs4 suppressor mutants (Fig. 5A and B). Igs4 displayed similar inclusion lysis phenotypes in OE129 and C57epi mouse oviduct epithelial cells, suggesting that the host defense program restricting Igs4 is also active in the cell type predominantly infected during genital C. muridarum infections (Fig. S3A and B) (37, 38).

10.1128/mBio.00385-19.3 FIG S3 Type I and type II interferons elicit lysis of Igs4 inclusions in mouse cells. IFN-γ and IFN-β inhibit Igs4 inclusion and progeny production in murine oviduct epithelial cells. Intact Igs4 inclusions (A) and progeny production (B) in OE129 and C57epi cells ± IFN-γ, ± IFN-β, or + or − CHX, and infected with identical inocula of Igs4. Graphs show results from three experiments performed in triplicate, and error bars show standard deviation. Samples were compared to untreated controls for each cell type (McCoy, OE129, or C57epi) by two-way ANOVA with Bonferroni posttest. *, *P* < 0.05; **, *P* < 0.01; ***, *P* < 0.001; ****, *P* < 0.0001. Download FIG S3, PDF file, 0.1 MB. Copyright © 2019 Giebel et al. 2019 Giebel et al. This content is distributed under the terms of the Creative Commons Attribution 4.0 International license.

We asked whether restriction of Igs4 required the induction of *de novo* host protein expression. Consistent with the involvement of host proteins in Igs4 inclusion lysis, the eukaryotic translation inhibitor cycloheximide (CHX) blocked inclusion lysis in untreated McCoy cells (Fig. 5C). We then tested if Igs4 was sensitive to interferon beta (IFN-β), because Chlamydia spp. are potent inducers of this cytokine, and some ISGs are cross-regulated by IFN-β and IFN-γ (39, 40). Exogenous IFN-β elicited excess Igs4 lysis that was blocked by an antibody that blocks IFN-β signaling through the type I interferon receptor (IFNAR) but not by an isotype control antibody (Fig. 5C). However, anti-IFNAR did not block Igs4 lysis in untreated McCoy cells, suggesting that Igs4 inclusion lysis in unprimed cells was IFNAR independent.

### IFN-γ kills Igs4-infected McCoy cells.

**FIG 6:**
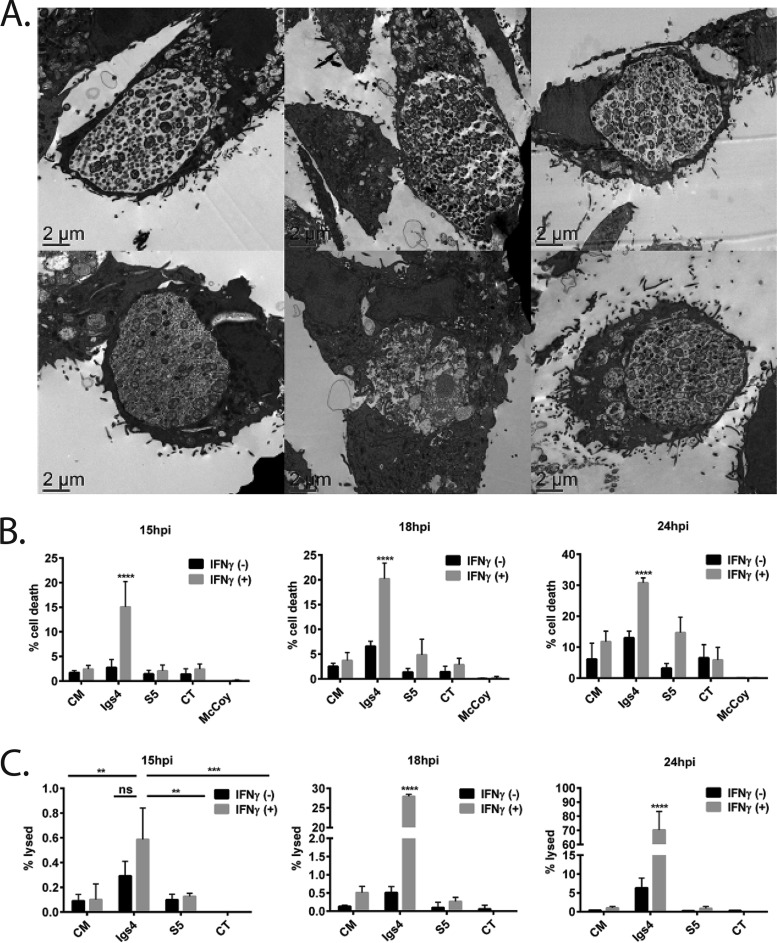
Igs4-infected IFN-γ-treated McCoy cells undergo increased cell death. (A) Representative TEM images of McCoy cells infected with *C. muridarum* (CM), Igs4, or S5 ± IFN-γ. (B) McCoy cells were infected with *C. muridarum*, Igs4, S5, or *C. trachomatis* (CT) at an MOI of 1 ± IFN-γ, and the percent dead McCoy cells was determined using acridine orange and ethidium bromide costaining at various intervals postinfection. (C) Same as in panel B, except the infections were performed at an MOI of 0.1, and lysed inclusions were identified by staining chlamydial LPS. Graphs in panels B and C show results from three experiments performed in triplicate, and error bars show standard deviation. Results with Igs4 and *C. muridarum* were compared to other conditions by two-way ANOVA with Bonferroni posttest. ns, not significant; ****, P < 0.0001.

Chlamydia-infected cells resist various inducers of programmed cell death (7), but transmission electron microscopy of Igs4-infected IFN-γ-primed McCoy cells revealed no intact inclusions and host cells that exhibited signs of cell death, including nuclear degradation, membrane blebbing, and increased intracellular vesicles (Fig. 6A). We tested if the McCoy cells were being killed using acridine orange/ethidium bromide costaining (41). More dead McCoy cells were detected in cultures infected with Igs4 than in cultures infected with C. muridarum, C. trachomatis, or the isogenic Igs4 suppressor mutant S5 (Fig. 6B). Priming with IFN-γ increased cell death in Igs4-infected McCoy cells at 16 hpi, corresponding with increased inclusion lysis, suggesting that inclusion lysis and host cell death occur in close succession (Fig. 6B and C).

### IFN-γ-mediated lysis of Igs4-infected cells is blocked by caspase inhibitors.

**FIG 7:**
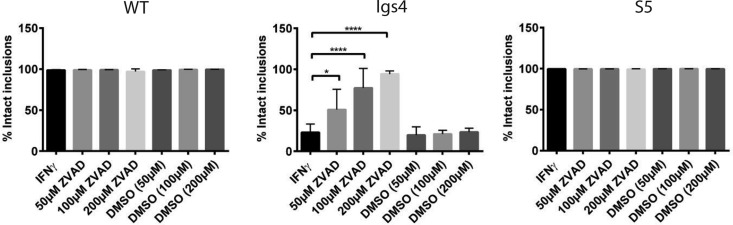
Lysis of Igs4 inclusions can be blocked by caspase inhibitors. McCoy cells plus IFN-γ were treated with various concentrations of Z-VAD (OMe)-fmk in DMSO or DMSO vehicle alone for 2 h and then infected with *C. muridarum* (CM), Igs4, or *C. trachomatis* (CT) at an MOI of 0.1. The infected cells were incubated with the same inhibitors (indicated below graphs) and IFN-γ until intact inclusions were counted at 24 hpi. Results from three experiments performed in triplicate are shown, error bars indicate standard deviation, and the results were analyzed by two-way ANOVA with Šidák’s multiple comparison. *, P < 0.05; ****, P < 0.0001. WT, wild type.

Necrostatin-1, an receptor interacting protein 1 (RIP-1) kinase inhibitor, blocked necrotic killing of McCoy cells induced by a combination of tumor necrosis factor-alpha (TNF-α) and the pancaspase inhibitor Z-VAD(OMe)-fmk (ZVAD) (Fig. S4A) (42, 43). However, necrostatin-1 failed to block lysis of Igs4 inclusions in IFN-γ-treated cells (Fig. S4B). In contrast, Z-VAD inhibited Igs4 lysis in IFN-γ-treated cells in a dose-dependent manner, and Igs4 lysis was completely blocked by 200 µM ZVAD (Fig. 7). These results suggested that Igs4 inclusion lysis was mediated by caspases (44).

10.1128/mBio.00385-19.4 FIG S4 Igs4 is not rescued by a necrosis inhibitor. (A) McCoy cell death induced by a necrotic stimulus [TNF-α plus Z-VAD(OMe)-fmk] can be reversed by necrostatin-1. McCoy cells were treated with 50 µM necrostatin-1 or DMSO control for 2 h and then were treated with 5 ng/ml TNF-α plus 10 µM Z-VAD (OMe)-fmk or DMSO control for 4 h. Cells were stained at 24 hpi with acridine orange/ethidium bromide and imaged. (B) Necrostatin-1 does not block Igs4 inclusion lysis elicited by IFN-γ. McCoy cells were pretreated for 2 h prior to infection with 50 µM necrostatin-1 in DMSO or DMSO alone and then were infected with *C. muridarum* (CM), Igs4, or S5 ± IFN-γ at an MOI of 0.1. The infected cells were fixed at 24 hpi and labeled with anti-LPS antibody. The percent intact Igs4 inclusions was compared to *C. muridarum* and S5 by two-way ANOVA with Šidák’s multiple-comparison test. ****, *P* < 0.0001. Error bars show standard deviation. Download FIG S4, PDF file, 0.4 MB. Copyright © 2019 Giebel et al. 2019 Giebel et al. This content is distributed under the terms of the Creative Commons Attribution 4.0 International license.

Because Z-VAD can have off-target effects, we tested if caspase-specific inhibitors rescued Igs4 lysis (44). Caspase-3 (Z-DEVD-FMK), caspase-8 (Z-IETD-FMK), and caspase-9 (Z-LEHD-FMK) inhibitors partially blocked IFN-γ-mediated Igs4 lysis (Fig. S5), suggesting that several caspases contribute to, but are not sufficient to elicit, Igs4 lysis (45–47). Unexpectedly, the levels of processed caspases-3, -8, and -9 were low in C. muridarum- and Igs4-infected cells in the presence and absence of IFN-γ (Fig. S6). These results suggested that Igs4 inclusion integrity is sensitive to small amounts of active caspases or that atypical caspases or other cysteine proteases mediate Igs4 lysis (48, 49).

10.1128/mBio.00385-19.5 FIG S5 Lysis of Igs4 inclusions is blocked by inhibitors of specific caspases. McCoy cells plus IFN-γ were treated with 200 µM Z-VAD (OMe)-fmk, 100 µM Z-DEVD-FMK (caspase-3 inhibitor), 100 µM Z-IETD-FMK (caspase-8 inhibitor), 100 µM Z-LEHD-FMK (caspase-9 inhibitor), or DMSO vehicle alone for 2 h and then infected with *C. muridarum* (CM), Igs4, or S5 at an MOI of 0.1. The infected cells were incubated with the same inhibitors and IFN-γ until intact inclusions were counted at 24 hpi. Results shown are from three experiments performed in triplicate, error bars indicate standard deviations, and the results were analyzed by two-way ANOVA with Šidák’s multiple-comparison test. *, *P* < 0.05; ***, *P* < 0.001; ****, *P* < 0.0001. Download FIG S5, PDF file, 0.1 MB. Copyright © 2019 Giebel et al. 2019 Giebel et al. This content is distributed under the terms of the Creative Commons Attribution 4.0 International license.

10.1128/mBio.00385-19.6 FIG S6 Caspase Western blot of Igs4-infected cells. (A) McCoy cells were mock, *C. muridarum* (CM), Igs4, or S5 infected at an MOI of 1 ± IFN-γ, and 1 µM staurosporine (stauro) was added to some wells at 20 hpi. The infected cells were lysed 24 hpi and samples and probed with pro- or cleaved caspase-3, caspase-8, or caspase-9 antibodies. Blots were imaged for the same exposure time (5 min) and full-length and cleaved caspase images (indicated by Cs) are from one blot. (B) Quantification of cleaved caspase-8 and caspase-9 in staurosporine infections from three Western blot experiments. In both cases, the *y* axis shows the levels of the cleaved caspases normalized to the levels in staurosporine-treated mock-infected cells. Graphs show the averages of the results from three experiments, and the error bars show standard deviation. Results from the three conditions were compared by two-way ANOVA with Bonferroni posttest. *, *P* < 0.05; ****, *P* < 0.0001. Download FIG S6, PDF file, 0.2 MB. Copyright © 2019 Giebel et al. 2019 Giebel et al. This content is distributed under the terms of the Creative Commons Attribution 4.0 International license.

### Apoptosis inducers elicit Igs4 lysis in the absence of IFN-γ.

**FIG 8:**
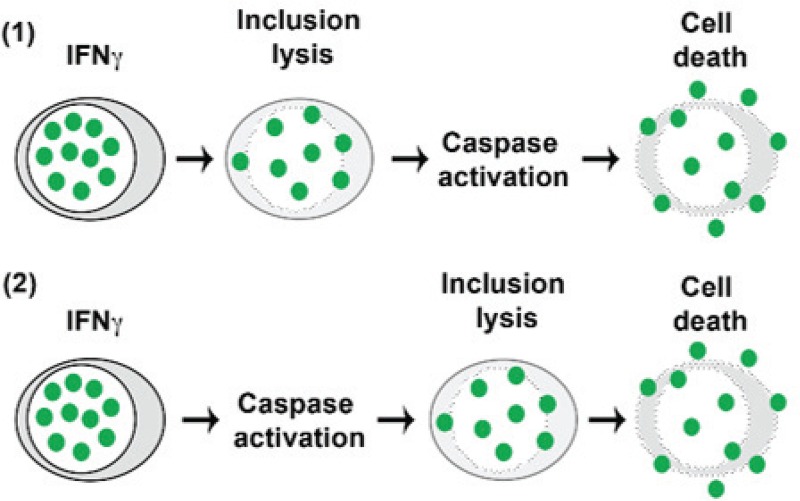
Two potential routes of Igs4 inclusion lysis and host cell death. In model 1, IFN-γ activates unknown membranolytic factors that disrupt Igs4 inclusions. Introduction of the inclusion contents into the cytoplasm then elicits activation of caspases that elicit host cell killing. In model 2, infection and IFN-γ are sufficient to elicit activation of caspases or other cysteine proteases that lyse Igs4 inclusions. Introduction of the inclusion contents into the cytoplasm elicits host cell death.

**FIG 9:**
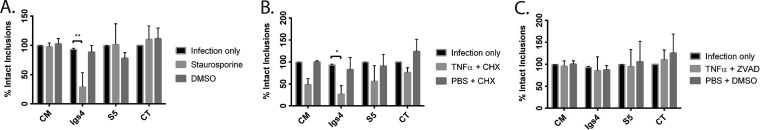
Inducers of apoptosis, but not necrosis, can elicit lysis of Igs4 inclusions. McCoy cells were infected at an MOI of 0.1 with *C. muridarum* (CM), Igs4, S5, or *C. trachomatis* (CT). At 20 hpi, 1 µM staurosporine (A), 5 ng/ml TNF-α plus 10 µg/ml CHX (B), 5 ng/ml TNF-α plus 10 µM Z-VAD (OMe)-fmk (C), or vehicle was added, or the cells were left untreated. The cells were fixed 4 h later, and the percent lysed inclusions was determined. Graphs in panels A to C show the results fom three experiments performed in triplicate, error bars indicate standard deviation, and the results were analyzed by two-way ANOVA with Šidák’s multiple comparison. *, P < 0.05; **, P < 0.005.

Our results suggested that IFN-γ-mediated lysis of Igs4 was mediated by capsases and or other cysteine proteases but not how these were activated. We hypothesized that caspase activation could result from the release of chlamydial antigens following inclusion lysis, or activation might precede and drive Igs4 lysis (Fig. 8). To determine whether caspase activation is sufficient to drive Igs4 inclusion lysis, we tested if apoptosis inducers lysed Igs4 inclusions in the absence of IFN-γ. Both staurosporine, an intrinsic apoptosis inducer, alone and TNF-α plus CHX, an extrinsic apoptosis inducer, lysed Igs4 in the absence of IFN-γ (Fig. 9A and B). Neither treatment lysed C. muridarum, C. trachomatis, or S5 inclusions. In contrast, a necrosis inducer, TNF-α plus Z-VAD, did not lyse Igs4 inclusions (Fig. 9C). These results suggested that Igs4 inclusions are sensitive to caspase-mediated rupture (Fig. 8).

### Igs4 attenuation in the murine genital tract is IFN-γ dependent.

**FIG 10:**
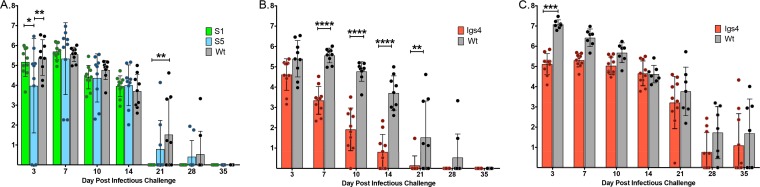
Igs4 attenuation in C57BL/6 is reversed in *IFN-γ ^−/−^* mice. (A) Vaginal EB shedding from C57/B6 mice infected with *C. muridarum* (wt), S1, or S5. (B) Vaginal EB shedding from C57/B6 mice infected with *C. muridarum* or Igs4. (C) Vaginal EB shedding from *IFN-γ ^−/−^* mice infected with Igs4 or *C. muridarum*. EB data are presented for individual mice and as mean (log_10_) inclusion-forming unit shedding ± the standard deviation. The data presented in panels A and B were collected from concurrently infected mice, and the *C. muridarum* infection data are presented in both panels for clarity. *, P < 0.05; **, *P* < 0.01; ***, P < 0.001; ****, P < 0.0001.

To determine the physiological impact of this novel IFN-γ-inducible membranolytic host defense pathway, we evaluated Igs4 virulence in a murine genital tract model. Infection duration and EB shedding were similar in C57BL/B6 mice infected with C. muridarum or with two different suppressor mutants isogenic to Igs4 (S1 and S5), confirming that background mutations in Igs4 do not impact its virulence (Fig. 10A). In contrast, wild-type mice cleared Igs4 earlier and shed fewer infectious EBs (Fig. 10B). Igs4 EB shedding decreased sharply 7 to 10 days postinfection (dpi), which is when CD4^+^ T cells begin to emigrate to the genital tract and shortly after IFN-γ levels peak in this model (50–52) (Fig. 10B). Igs4-infected mice also did not develop hydrosalpinx (0/10), whereas most mice infected with C. muridarum or the suppressor mutants did (C. muridarum, 9/9 mice; S1, 10/10 mice; S5, 7/9 mice). Attenuation of Igs4 was IFN-γ dependent. The duration and magnitude of Igs4 shedding in *IFN-γ* ^−/−^ mice did not significantly differ from C. muridarum from mid-late infection onwards (Fig. 10C). Notably, Igs4-infected *IFN-γ* ^−/−^ mice shed fewer EBs than did C. muridarum-infected *IFN-γ* ^−/−^ mice at 3 dpi, when type I interferon levels are high in this model (53, 54). Igs4 EB shedding also trended lower until 10 dpi, possibly reflecting the switch from a type I to a type II interferon-dominated environment. Collectively, these data indicate that an IFN-γ-driven and cysteine protease-dependent host defense program, resulting in inclusion lysis, potently sterilizes genital Chlamydia infections *in vivo*.

## DISCUSSION

Chlamydial interferon evasion is key to the virulence of multiple Chlamydia spp. in mammals (51, 53, 55, 56), but few genes that contribute to chlamydial interferon resistance have been characterized (28, 57, 58). We identified 31 Igs C. muridarum mutants using a genetic screen. The Igs mutants exhibited an array of phenotypes and contained few mutations in the same genes, indicating that C. muridarum IFN-γ resistance is a complex phenotype (Fig. 1 and Table S1). This result may reflect inability of a single chlamydial effector to counteract mechanistically distinct IFN-γ-mediated cell-autonomous defenses and the importance of IFN-γ resistance in C. muridarum virulence.

We focused our follow-up studies on Igs4, which showed a reproducible phenotype with a substantial effect size. To identify the causative mutation in Igs4, we conducted a suppressor screen and found that several Igs4 suppressor mutants had mutations that inactivate TC0574, indicating that *tc0574* is dispensable for chlamydial IFN-γ and apoptosis resistance and that the specific *tc0574* mutation found in Igs4 (TC0574^G81E^) rendered the mutant highly sensitive to an IFN-γ-inducible cell-intrinsic immune program. How this single amino acid change in TC0574 confers IFN-γ sensitivity remains unclear.

TC0574 is predicted to be an Inc based upon the presence of a hydrophobic bilobed domain (59, 60). Similar to a prior study that attempted to localize the C. trachomatis TC0574 ortholog (CT0300) (61), we were unable to confirm inclusion membrane localization of TC0574. A Phyre2 model predicts that TC0574 contains two tightly packed α-helices (amino acids [aa] 41 to 63 and aa 76 to 98) and that G81 locates to the interface of these helices (Fig. S7) (62). The core region between two similar α-helices in IncA regulates homodimer formation and can block soluble *N*-ethylmaleimide-sensitive factor attachment receptor (SNARE)-mediated and homotypic inclusion fusion (63). We speculate that disruption of TC0574 folding alters interactions with other proteins that mediate resistance of C. muridarum inclusions to cysteine proteases.

10.1128/mBio.00385-19.7 FIG S7 *Ab initio* modeling and alignment of *C. muridarum* TC0574 and homologs. (A) Putative TC0574 structure was made using the Phyre2 algorithm and manipulated in PyMOL. The N terminus of the protein is colored orange, and the C terminus is colored green. G81 is found at the intersection of the two α-helices, which are predicted to be tightly packed together. The G81E mutation in Igs4 could disrupt tight packing of the two α-helices and therefore disrupt proper protein folding. (B) Alignment of TC0574 and its homologs in *C. muridarum* Nigg (Cm), *C. muridarum* serovar D (Ct), and *Chlamydia suis* MD56 (Cs) utilizing Clustal Omega. Conserved residues are indicated below the sequence, where an asterisk indicates a single fully conserved residue, a colon indicates conservation of amino acids with strong similarities, and a period indicates conservation of amino acids with weak similarities. Each homolog contains a GGLG conserved motif from aa 80 to 83 (based on TC0574 sequence). The Igs4 TC0574^G81E^ mutation lies within this highly conserved GGLG motif. Download FIG S7, PDF file, 0.4 MB. Copyright © 2019 Giebel et al. 2019 Giebel et al. This content is distributed under the terms of the Creative Commons Attribution 4.0 International license.

Chlamydia-infected cells resist apoptosis inducers, including staurosporine (kinase inhibitor), etoposide (DNA-damaging agent), TNF-α, Fas antibody, granzyme, and perforin (7). Chlamydia PCDR has been associated with inhibition of the proapoptotic proteins Bax and Bak, upregulation and stabilization of the antiapoptotic protein Mcl1, upregulation and translocalization of hexokinase II to the mitochondrion, and blocking internalization of TNF-α-TNFR1 complexes (7, 8, 64–69). Broadly, chlamydial apoptosis resistance appears to occur upstream of mitochondrial membrane destabilization and the release of inner membrane components. Sensitivity of Igs4-infected cells to staurosporine, and to TNF-α plus CHX, which activate caspase-3 and -8, respectively, implies that Igs4 cannot inhibit upstream or downstream steps in the caspase cascade. Type I and II interferons increase procaspase-8 expression and synergize with TNF-α to trigger FADD-mediated procaspase-8 processing (70), so TNF-α plus CHX and interferon may elicit lysis of Igs4 inclusions by this pathway. Blockage of IFN-γ-mediated Igs4 inclusion lysis by ZVAD and its partial inhibition by individual caspase inhibitors suggest the contribution of multiple caspases but do not clarify which caspases drive inclusion lysis or exclude the involvement of other cysteine proteases, such as calpains, which promote lysis of C. trachomatis inclusions triggered by laser ablation (71). Our failure to detect increased levels of caspases in the absence of staurosporine could indicate that classical initiator and executioner caspases are not major mediators of Igs4 lysis or could reflect exquisite sensitivity of the Igs4 inclusion to low levels of active caspases (Fig. S6).

Inactivation of some Incs elicits inclusion instability in diverse cell lines and in the absence of specific stimuli. It was proposed that the inclusion lysis phenotype of a chlamydial promoter of survival (CpoS) mutant indicated a specific role for CpoS in shielding host cells from prodeath signals and STING-mediated induction of type I interferons (24). Another study showed that inactivation of other Incs (CT0229, IncC, and CT0383) destabilizes the inclusion and suggested that STING activation is a common endpoint of release of inclusion contents into the cytosol (25). Lysis of CpoSs, IncC, CT0229, and CT0383 mutant inclusions is insensitive to caspase inhibitors, arguing that caspase activation in cells infected with these mutants is a consequence, and not a cause, of inclusion lysis (24, 25). When C. trachomatis inclusions were lysed using laser ablation, their host cells were rapidly killed via an undefined necrotic pathway that does not require BAX, BAK, RIP-1, or caspases (71). High doses of TNF-α plus CHX can also elicit necrotic killing of C. trachomatis-infected cells via a noncanonical pathway that is caspase-8 dependent (72). Interpreting these observations in the context of our results, we hypothesize that Chlamydia spp. employ effectors that counter cytokine-mediated activation of caspases, but that release of chlamydial antigens following loss of inclusion integrity invariably leads to initiation of a poorly defined pathway of programmed cell death (Fig. 8).

In conclusion, Chlamydia-infected cells exhibit profound PCDR. Based upon our observations that Igs4 responds similarly to interferon and nonimmune apoptotic stimuli in mouse cells, that Igs4 inclusion rupture is blocked by cysteine protease inhibitors, and that Igs4 virulence is rescued in *IFN-γ^−/−^* mice, we propose that Chlamydia spp. evolved resistance to IFN-γ-inducible caspase activation primarily to avoid the lytic destruction of its inclusions. Thus, chlamydial PCDR may have evolved as a serendipitous by-product of a strategy by Chlamydia spp. to protect the integrity of its inclusion. Igs4 could be a powerful tool to investigate the role of caspase-mediated cell-autonomous immune responses and programmed cell death pathways in Chlamydia pathogenesis using highly tractable mouse models.

## MATERIALS AND METHODS

### Chlamydia propagation and cell culture.

C. muridarum and C. trachomatis serovar L2 strain 434/Bu were gifts from Harlan Caldwell, NIH-NIAID, and a C. trachomatis serovar L2 strain 434/Bu rifampin-resistant (rif^r^) isolate (C. trachomatis rif^r^) was described previously (33). Chlamydia spp. were propagated in McCoy or HeLa cells, and EBs were purified by density gradient centrifugation (73). OE129 and C57epi cells were gifts from Wilbert Derbigny and Ray Johnson. Dulbecco’s modified Eagle’s medium (HyClone) supplemented with 10% fetal bovine serum, sodium pyruvate (Atlanta Biologicals), nonessential amino acids (Gibco), and 5 µM HEPES buffer (Sigma) (DMEM) was used for cell culture and was supplemented with mouse recombinant IFN-γ (R&D Systems).

### Library construction and Igs mutant screen.

The C. muridarum mutant library was constructed using 15 mg/ml EMS, similarly to what we described previously (27, 28). Library isolates were plaque-cloned and then lysed by bead agitation (74). McCoy cell monolayers in 96-well plates were incubated in fresh DMEM with or without 20 U IFN-γ for 24 h, infected with equal inocula of library lysates, and methanol fixed at 24 hpi. Inclusions were labeled with a murine monoclonal antibody against chlamydial LPS (EVI-H1), a secondary Alexa488-conjugated antibody (Life Technologies), and imaged using an EVOS FL auto cell imaging microscope. For inclusion assays, cells were fixed in methanol, and inclusions were antibody labeled. For progeny assays, cells were harvested by scraping and EBs released by bead agitation. Dilutions were used to infect McCoy cells, and inclusions were counted at 24 hpi.

### Inclusion morphology.

McCoy cells were grown on glass coverslips and infected by centrifugation and rocking. The cells were fixed at 24 hpi, labeled with Evans blue, and then labeled with EVI-HI and secondary antibody. Coverslips were mounted in Prolong gold antifade reagent with 4′,6-diamidino-2-phenylindole (DAPI; Life Technologies) and imaged using a Leica DMI6000B microscope.

### IFN-β and IFNAR-1 and coinfection rescue experiments.

McCoy monolayers were incubated with 10 µM anti-mouse IFNAR-1 antibody (clone MAR-5A3), an IgG1 isotype control antibody (BioLegend), or IFNAR-1 antibody and 250 U/ml IFN-β (R&D Systems) for 1 h, infected with C. muridarum or Igs4, and fixed at 24 hpi. Coinfections of McCoy cells with C. trachomatis rif^r^ and C. muridarum or Igs4 were performed as described previously (33).

### C. muridarum genome sequencing.

Purified DNA from EBs was treated with NEBNext double-stranded DNA (dsDNA) Fragmentase (NEB) to generate dsDNA fragments. The TruSeq Nano DNA sample preparation kit was used to prepare sequencing libraries as per the manufacturer’s protocols (Illumina, Inc.). Samples were multiplexed and paired-end 100-bp sequenced on an Illumina HiSeq 2500 platform.

### Isolation of Igs4 suppressor mutants and Igs4 recombinants.

IFN-γ-treated McCoy cells were infected with Igs4 and incubated for 24 h with or without IFN-γ. The cells were harvested, a fifth of each harvest was used to infect new IFN-γ-treated McCoy monolayers, and the entire process was repeated six times. Cells in 23 out of 96 experiments were heavily infected by passage six, and suppressor isolates were plaque-cloned from these experiments. For isolation of Igs4 recombinants, IFN-γ-treated McCoy cells were coinfected with Igs4 and a temperature-sensitive C. muridarum isolate identified by screening the mutant library for isolates that grew at 37°C but not 40°C (27). Lysates from the coinfection were passed twice in IFN-γ-treated McCoy cells incubated at 40°C, and then the surviving isolates were plaque-cloned.

### Irgb6 and Irgb10 localization.

McCoy cells in DMEM were treated overnight with IFN-γ or left untreated and were infected at a multiplicity of infection (MOI) of ∼1 by centrifugation. Thirty minutes postcentrifugation, medium with or without IFN-γ was added. The cells were washed with phosphate-buffered saline (PBS) at 22 hpi, fixed with cold methanol, washed with PBS, and then blocked for 30 min at room temperature in buffer containing 5% goat serum and 5% bovine serum albumin (BSA). Cells were incubated with EVI-H1, rabbit polyclonal anti-Irgb6 antiserum, or rabbit polyclonal anti-Irgb10 antiserum and stained with Alexa Fluor-conjugated secondary antibodies (Molecular Probes) and Hoescht (15, 32). The percentage of targeted inclusions was determined as a function of the number of Irgb6- or Irgb10-positive inclusions divided by the total number of inclusions.

### Evaluation of bacterial burden in MEFs.

Wild-type, *irgm1*^−/−^ or *irgm3*^−/−^, and *atg5*^−/−^ MEFs were cultured in DMEM and treated overnight with IFN-γ or left untreated. MEFs were infected at an MOI of ∼1 by centrifugation, and then medium with or without IFN-γ was added. The cells were lysed in 400 μl H_2_O at 37°C for 10 min before the addition of 100 μl 5× 250 mM sucrose–10 mM sodium phosphate–5 mM l-glutamic acid pH 7.2 (1× SPG) at 30 hpi. Relative progeny was determined based on infectivity in untreated McCoy cells.

### shRNAs.

MEFs were transduced with The RNAi Consortium (TRC) shRNA vectors. Lentivirus was prepared in HEK293T cells. Cells were selected for expression for a minimum of 48 h with puromycin (5 μg/ml). Knockdown efficiency of >70% was confirmed for each construct using quantitative (qPCR). TRC shRNA vectors TRCN0000251780 and TRCN0000251781 were used.

### Griess assay and Western blotting of iNOS expression.

Nitrite was measured using a commercial Griess assay kit, according to the manufacturer’s instructions. Cells from Griess assay experiments were used in Western blotting experiments, and the proteins were labeled with anti-iNOS monoclonal antibody (MAb) (no. 2982S) or anti-murine glyceraldehyde-3-phosphate dehydrogenase (GAPDH) MAb (no. 2118S), followed by a horseradish peroxidase-conjugated MAb antibody (no. 7074; Cell Signaling), and visualized using SuperSignal West Pico chemiluminescent substrate (Pierce).

### Transmission electron microscopy.

Cells were fixed with 2.5% glutaraldehyde and 4% formaldehyde in PBS for 1 h and then rinsed 3× with 0.1 M sodium cacodylate buffer. Cells were postfixed in 1% osmium tetroxide and 1% tannic acid, dehydrated, and embedded in epoxy resin. Sections were stained with uranyl acetate and lead citrate and imaged using a JEOL JEM 1011 microscope with a Gatan 890 4k by 4k digital camera.

### Apoptosis and necrosis inducer and inhibitor experiments.

McCoy monolayers were treated with various concentrations of Z-VAD(OMe)-fmk (Santa Cruz Biotechnology), 50 µM necrostatin-1 (Santa Cruz Biotechnology), 100 µm of Z-DEVD-FMK, Z-IETD-FMK, or Z-LEHD-FMK (R&D Systems, Inc.), or dimethyl sulfoxide (DMSO) vehicle alone for 2 h prior to infection. Cells were infected at an MOI of 0.1, and IFN-γ was added in some cases. For inducer experiments, McCoy cells were infected at an MOI of 0.1 (EVI-H1 staining) or at an MO1 of 1 (acridine orange/ethidium bromide staining). At 20 hpi, 1 µM staurosporine (Santa Cruz Biotechnology), 5 ng/ml TNF-α (BioLegend-recombinant mouse) and 10 µg/ml CHX, or 5 ng/ml TNF-α and 10 µM ZVAD(OMe)-fmk was added. Cells were stained with acridine orange and ethidium bromide at 24 hpi or labeled with EVI-H1 as described above. To assay cell death, 4 µl of 100 µg/ml acridine orange (Sigma) and ethidium bromide (Sigma) were added to DMEM and incubated 15 min at room temperature (RT) (41). To assay inclusion lysis, cells were fixed with methanol, labeled with EVI-H1, and imaged as described above.

### Mouse genital tract infections.

Six 10-week-old female C57BL/6J or B6.129S7-IFN-γ^tm1Ts^/J (*IFN-γ* ^−/−^ mice) (Jackson Laboratories, Bar Harbor, ME) were treated with 2.5 mg Depo-Provera at 10 and 3 days prior to infection. Mice were inoculated with C. muridarum or mutants by placing 5 µl of SPG containing 50,000 inclusion-forming units into the vaginal vault. The vaginal vault was swabbed (days 3, 7, 10, and 14 and weekly thereafter), and the infections were monitored by counting inclusions (75).

Analysis of variance (ANOVA) models were used to (i) test whether the time trends between treatment groups were parallel and, if they were parallel, and (ii) determine whether the groups differ with respect to magnitude of response. An α level of 0.05 was deemed significant.
