# Supplementary figures and images for: Genetic Screen in Chlamydia muridarum Reveals Role for an Interferon-Induced Host Cell Death Program in Antimicrobial Inclusion Rupture
Source: mBio. 2019 Apr 9;10(2):e00385-19. doi: 10.1128/mBio.00385-19 (PMC6456753; doi:10.1128/mBio.00385-19)

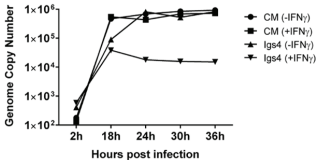

Supplement: FIG S1 [file mBio.00385-19-sf001.pdf]

A.

|        | CM | Igs4 | R3 | R5 | R7 |
|--------|----|------|----|----|----|
| TC0094 | S  | F    | S  | F  | F  |
| TC0157 | V  | A    | V  | A  | A  |
| TC0431 | D  | N    | D  | N  | N  |
| TC0433 | S  | F    | S  | F  | F  |
| TC0462 | S  | N    | S  | N  | N  |
| TC0574 | G  | E    | G  | G  | G  |
| TC0610 | G  | E    | G  | E  | G  |
| TC0741 | M  | I    | I  | I  | I  |
| TC0769 | S  | F    | F  | F  | F  |

B.

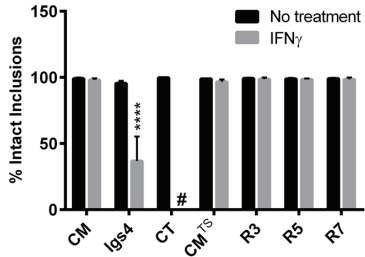

C.

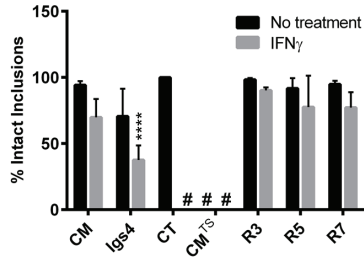

Supplement: FIG S2 [file mBio.00385-19-sf002.pdf]

A.

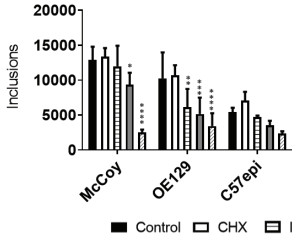

B.

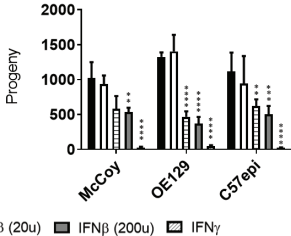

Supplement: FIG S3 [file mBio.00385-19-sf003.pdf]

A.

TNF $\alpha$  + ZVADTNF $\alpha$  + ZVAD  
+ necrostatin-1

DMSO

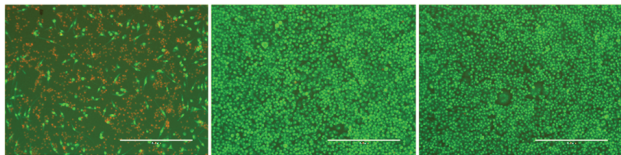

B.

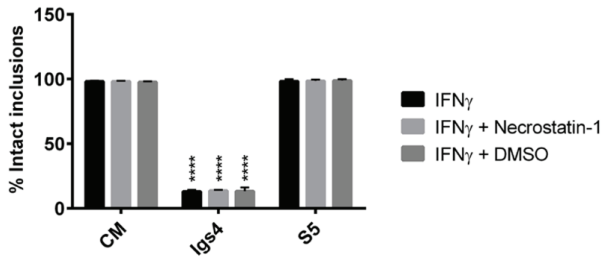

Supplement: FIG S4 [file mBio.00385-19-sf004.pdf]

CM

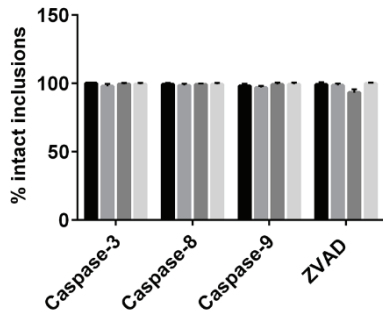

Igs4

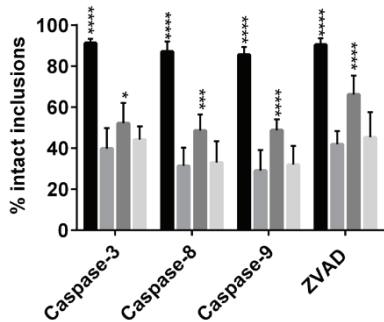

S5

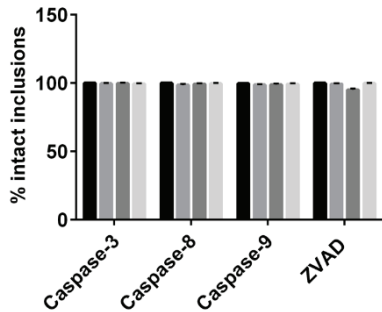

No treatment

IFN $\gamma$ IFN $\gamma$  + 100 $\mu$ MIFN $\gamma$  + DMSO

Supplement: FIG S5 [file mBio.00385-19-sf005.pdf]

A.

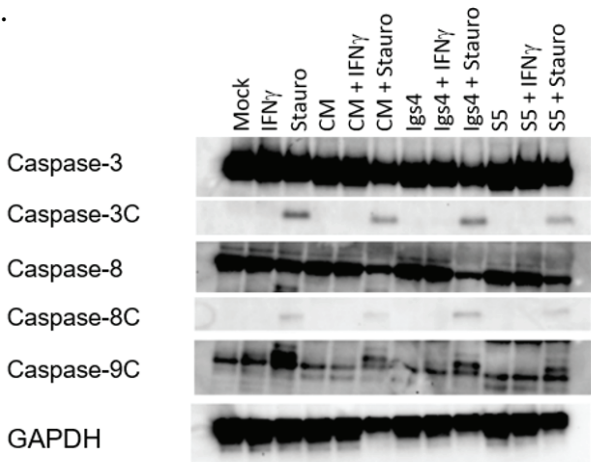

B.

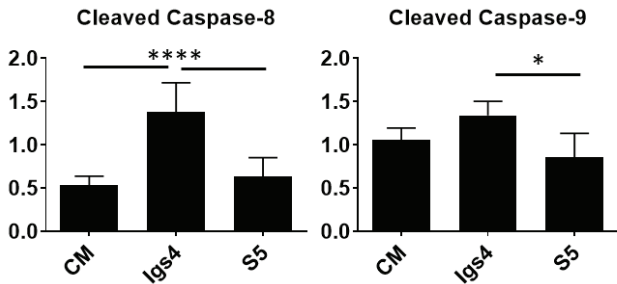

Supplement: FIG S6 [file mBio.00385-19-sf006.pdf]
