# Supplementary material for: Genetic Screen in Chlamydia muridarum Reveals Role for an Interferon-Induced Host Cell Death Program in Antimicrobial Inclusion Rupture
Source: mBio. 2019 Apr 9;10(2):e00385-19. doi: 10.1128/mBio.00385-19 (PMC6456753; doi:10.1128/mBio.00385-19)
Supplement: TABLE S1 [file mBio.00385-19-st001.docx]

**Table S1. SNPs in the genomes of IGS mutants.**

| **Igs1** |  |  |  |  |
| --- | --- | --- | --- | --- |
| **Gene ID** | **Description** | **Nucleotide Position** | **Nucleotide Change** | **Amino Acid Change** |
| *tc0004* | Transcription elongation protein, GreA/GreB family | 3413 | C -> T | Pro -> Ser |
| *tc0090* | Virulence ATPase, putative | 108613 | G -> A | Leu -> Phe |
| *tc0180* | CDP-diacylglycerol-glycerol-3-phosphate 3-phosphatidyltransferase | 214411 | G -> A | Leu -> Leu |
| - | Intergenic | 227381 | G -> A | - |
| *tc0243* | ABC transporter, permease protein, putative | 281925 | C -> T | Asp -> Asn |
| *tc0256* | Conserved hypothetical protein | 299426 | C -> T | Pro -> Pro |
| *tc0271* | Glutamyl-tRNA (gln) amidotransferase subunit A (gatA) | 325196 | G -> A | Met -> Ile |
| *tc0320* | Hypothetical protein | 377362 | G -> A | Thr -> Ile |
| *tc0412* | Conserved hypothetical protein | 472952 | C -> T | Gln -> stop |
| *tc0439* | Adherence factor | 532334 | C -> T | Ala -> Val |
| *tc0851* | Type III secretion inner membrane protein, sctR | 987831 | C -> T | Ala -> Val |
| *tc0862* | Conserved hypothetical protein | 997718 | G -> A | Arg -> Cys |
| *tc0868* | Conserved hypothetical protein | 1005383 | G -> A | Gly -> Arg |
| **Igs2** |  |  |  |  |
| **Gene ID** | **Description** | **Nucleotide Position** | **Nucleotide Change** | **Amino Acid Change** |
| *tc0008* | Exodeoxyribonuclease V, gamma subunit, putative | 13782 | G-> A | Leu -> Leu |
| *tc0052* | Major outer membrane protein, porin (ompA) | 58829 | T -> C | Lys -> Glu |
| *tc0056* | Conserved hypothetical protein | 66042 | G -> A | Val -> Ile |
| *tc0197* | polymorphic membrane protein D family (pmpD) | 230212 | G -> A | Gln -> Gln |
| *tc0412* | Conserved hypothetical protein | 473769 | T -> G | Leu -> Stop |
| *tc0476* | Sodium:sulfate symporter family protein | 577763 | G -> A | Ser -> Asn |
| *tc0481* | Leucyl-tRNA synthetase (leuS) | 585522 | G -> A | Phe -> Phe |
| *tc0496* | Hypothetical protein | 603314 | G -> A | Ser -> Ser |
| *tc0671* | Conserved hypothetical protein | 800632 | G -> A | Ser -> Leu |
| *tc0726* | Sulfur rich protein, srp | 864375 | C -> T | Gly -> Arg |
| *tc0820* | UDP-3-0-(3-hydroxymyristoyl) N-acetylglucosamine deacetylase (lpxC) | 952912 | C -> T | Glu -> Lys |

| **Igs3** |  |  |  |  |
| --- | --- | --- | --- | --- |
| **Gene ID** | **Description** | **Nucleotide Position** | **Nucleotide Change** | **Amino Acid Change** |
| *tc0090* | Virulence, ATPase putative | 108613 | G -> A | Leu -> Phe |
| *tc0143* | UDP-N-acetylmuramate--alanine ligase/D-alanine--D-alanine ligase (murC/ddlA) | 174709 | C -> T | Ser -> Leu |
| *tc0243* | ABC transporter, permease putative | 281925 | C -> T | Asp -> Asn |
| *tc0303* | Conserved hypothetical protein | 361136 | G -> A | Asn -> Asn |
| *tc0412* | Conserved hypothetical protein | 472952 | C -> T | Gln -> Stop |
| *tc0414* | Conserved hypothetical protein | 474871 | G -> A | Val -> Ile |
| *tc0476* | Sodium:sulfate symporter family protein | 578503 | C -> T | Gln -> Stop |
| *tc0799* | Ribosomal protein S5 | 940807 | C -> T | Val -> Val |

| **Igs4** |  |  |  |  |
| --- | --- | --- | --- | --- |
| **Gene ID** | **Description** | **Nucleotide Position** | **Nucleotide Change** | **Amino Acid Change** |
| *tc0094* | NifS-related protein | 112667 | G -> A | Ser -> Phe |
| *tc0157* | Aas bifunctional protein | 190522 | G -> A | Asp -> Asn |
| - | Intergenic | 216687 | G -> A | - |
| - | Intergenic | 303555 | C -> T | - |
| *tc0412* | Conserved hypothetical protein | 473353 | A -> T | Gln -> His |
| *tc0425* | Monooxygenase-related protein | 491571 | G -> A | His -> His |
| *tc0431* | MAC/perforin | 496343 | C -> T | Asp -> Asn |
| *tc0431* | MAC/perforin | 497583 | G -> A | Ser -> Ser |
| *tc0433* | Phospholipase D, pld | 500955 | G -> A | Ser -> Phe |
| *tc0462* | DNA gyrase, subunit B | 561752 | C -> T | Ser -> Asn |
| *-* | Intergenic | 565121 | G -> A | - |
| *-* | Intergenic | 647536 | C -> T | - |
| *tc0574* | Hypothetical protein | 684336 | G -> A | Gly -> Glu |
| *tc0610* | Excinuclease ABC, subunit A | 730959 | G -> A | Gly -> Glu |
| *tc0684* | Conserved hypothetical protein | 817790 | G -> A | Ser -> Ser |
| *tc0692* | Lipid A disaccharide synthase | 823967 | G -> A | Ala -> Ala |
| *tc0741* | TARP | 886389 | G -> A | Met -> Ile |
| *tc0769* | Conserved hypothetical protein | 911628 | G -> A | Ser -> Phe |
| *tc0776* | Glucose-1-phosphate adenylytransferase, glgC | 918129 | C -> T | Leu -> Leu |

| **S1** |  |  |  |  |
| --- | --- | --- | --- | --- |
| **Gene ID** | **Description** | **Nucleotide Position** | **Nucleotide Change** | **Amino Acid Change** |
| *tc0094* | NifS-related protein | 112667 | G -> A | Ser -> Phe |
| *tc0157* | Aas bifunctional protein | 190522 | G -> A | Asp-> Asn |
| - | Intergenic | 216687 | G -> A | - |
| - | Intergenic | 303555 | C -> T | - |
| *tc0412* | Conserved hypothetical protein | 473353 | A -> T | Gln -> His |
| *tc0425* | Monooxygenase-related protein | 491571 | G -> A | His -> His |
| *tc0431* | MAC/perforin | 496343 | C -> T | Asp -> Asn |
| *tc0431* | MAC/perforin | 497583 | G -> A | Ser -> Ser |
| *tc0433* | Phospholipase D, pld | 500955 | G -> A | Ser -> Phe |
| *tc0462* | DNA gyrase, subunit B | 561752 | C -> T | Ser -> Asn |
| - | Intergenic | 565121 | G -> A | - |
| - | Intergenic | 647536 | C -> T | - |
| *tc0574* | Hypothetical protein | 684336 | G -> A | Gly -> Glu |
| *tc0574* | Hypothetical protein | 684375 | C -> A | Thr -> Asn |
| *tc0610* | Excinuclease ABC, subunit A | 730959 | G -> A | Gly -> Glu |
| *tc0684* | Conserved hypothetical protein | 817790 | G -> A | Ser -> Ser |
| *tc0692* | Lipid A disaccharide synthase | 823967 | G -> A | Ala -> Ala |
| *tc0741* | TARP | 886389 | G -> A | Met -> Ile |
| *tc0769* | Conserved hypothetical protein | 911628 | G -> A | Ser -> Phe |
| *tc0776* | Glucose-1-phosphate adenylytransferase, glgC | 918129 | C -> T | Leu -> Leu |
| **S5** |  |  |  |  |
| **Gene ID** | **Description** | **Nucleotide Position** | **Nucleotide Change** | **Amino Acid Change** |
| *tc0094* | NifS-related protein | 112667 | G -> A | Ser -> Phe |
| *tc0157* | Aas bifunctional protein | 190522 | G -> A | Asp -> Asn |
| - | Intergenic | 216687 | G -> A | - |
| - | Intergenic | 303555 | C -> T | - |
| *tc0412* | Conserved hypothetical protein | 473353 | A -> T | Gln -> His |
| *tc0425* | Monooxygenase-related protein | 491571 | G -> A | His -> His |
| *tc0431* | MAC/perforin | 496343 | C -> T | Asp -> Asn |
| *tc0431* | MAC/perforin | 497583 | G -> A | Ser -> Ser |
| *tc0433* | Phospholipase D, pld | 500955 | G -> A | Ser -> Phe |
| *tc0462* | DNA gyrase, subunit B | 561752 | C -> T | Ser -> Asn |
| *-* | Intergenic | 565121 | G -> A | - |
| *-* | Intergenic | 647536 | C -> T | - |
| *tc0574* | Hypothetical protein | 684200 | C -> T | Gln -> Stop |
| *tc0574* | Hypothetical protein | 684336 | G -> A | Gly -> Glu |
| *tc0610* | Excinuclease ABC, subunit A | 730959 | G -> A | Gly -> Glu |
| *tc0673* | Heat shock gene repressor, HcrA | 804307 | C ->T | Ser -> Ser |
| *tc0684* | Conserved hypothetical protein | 817790 | G -> A | Ser -> Ser |
| *tc0692* | Lipid A disaccharide synthase | 823967 | G -> A | Ala -> Ala |
| *tc0741* | TARP | 886389 | G -> A | Met -> Ile |
| *tc0769* | Conserved hypothetical protein | 911628 | G -> A | Ser -> Phe |
| *tc0776* | Glucose-1-phosphate adenylytransferase, glgC | 918129 | C -> T | Leu -> Leu |
