# Supplementary material for: Genetic Screen in Chlamydia muridarum Reveals Role for an Interferon-Induced Host Cell Death Program in Antimicrobial Inclusion Rupture
Source: mBio. 2019 Apr 9;10(2):e00385-19. doi: 10.1128/mBio.00385-19 (PMC6456753; doi:10.1128/mBio.00385-19)
Supplement: FIG S7 [file mBio.00385-19-sf007.pdf]

A.

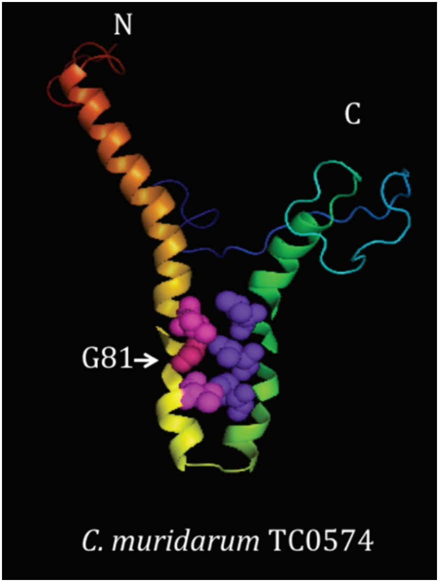

B.

```
Cm     MGNAFNNPFIFRDINMNNIT--LSASSSSLNSEPDRPQ-EKVNPHLLFILALIPVIGLGVALYLCIK   64
Ct     ------------MCYVLNFITGKYSTPSPNNCRPQSPQKTGGHSNLLFVLALLPVIGLGVAIYLCAR   55
Cs     ---------------MTQLISSTSSSSSVPHSEPFYPP-SKPHSSLLFVLAQLPIIGLAVACYLCVQ   51
                      : ::     :: *  ...*  *     .  ***:** :*:***.** *** :

Cm     TTDVHWKSNAGVAVLGGLGILAIAYILIQTLLVIRL----AFKKINNFLHHPTLPCCISFSCKTKTE   127
Ct     R-ANYWKGNAAIATAGGLGLITIAAALMVVITPIIFCLRY----LYQLLRQLPAHCCGSSHQIEI--   115
Cs     R-GPHWKSSAGVAIAGGLGLTAFAAVLIYTLGYAFMGIFYAIGLVAKNIHQRASSCRNPFLVEQ---   114
           :**..*.:*  ****: ::*  *: .:    :        : : :::
```
